# The rapid rise of SARS‐CoV‐2 Omicron subvariants with immune evasion properties: XBB.1.5 and BQ.1.1 subvariants

**DOI:** 10.1002/mco2.239

**Published:** 2023-03-15

**Authors:** Danyi Ao, Xuemei He, Weiqi Hong, Xiawei Wei

**Affiliations:** ^1^ Laboratory of Aging Research and Cancer Drug Target, State Key Laboratory of Biotherapy and Cancer Center, National Clinical Research Center for Geriatrics West China Hospital, Sichuan University Chengdu Sichuan China

**Keywords:** BQ.1 and XBB lineages, immune escape, Omicron, XBB.1.5

## Abstract

As the fifth variant of concern of the SARS‐CoV‐2 virus, the Omicron variant (B.1.1.529) has quickly become the dominant type among the previous circulating variants worldwide. During the Omicron wave, several subvariants have emerged, with some exhibiting greater infectivity and immune evasion, accounting for their fast spread across many countries. Recently, two Omicron subvariants, BQ.1 and XBB lineages, including BQ.1.1, XBB.1, and XBB.1.5, have become a global public health issue given their ability to escape from therapeutic monoclonal antibodies and herd immunity induced by prior coronavirus disease 2019 (COVID‐19) vaccines, boosters, and infection. In this respect, XBB.1.5, which has been established to harbor a rare mutation F486P, demonstrates superior transmissibility and immune escape ability compared to other subvariants and has emerged as the dominant strain in several countries. This review provides a comprehensive overview of the epidemiological features, spike mutations, and immune evasion of BQ.1 and XBB lineages. We expounded on the mechanisms underlying mutations and immune escape from neutralizing antibodies from vaccinated or convalescent COVID‐19 individuals and therapeutic monoclonal antibodies (mAbs) and proposed strategies for prevention against BQ.1 and XBB sublineages.

## Graphical abstract

In the Omicron wave, two emerging subvariants, BQ.1 and XBB, have rapidly become a global public health concern. This diagram illustrates the critical spike mutations in these subvariants, which can lead to immune escape from prior COVID‐19 vaccines, boosters, and therapeutic monoclonal antibodies (mAbs). Notably, XBB.1.5, which carries a rare F486P mutation, has demonstrated significantly higher receptor‐binding domain (RBD)‐ACE2 binding affinity and transmissibility compared to other subvariants, making it the dominant strain in several countries.

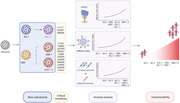

## 1 INTRODUCTION

The world has been facing the coronavirus disease 2019 (COVID‐19) pandemic for more than 3 years. To date, five SARS‐CoV‐2 variants, including Alpha, Beta, Gamma, Delta, and Omicron, have been categorized as variants of concern (VOCs). The fifth VOC, Omicron (B.1.1.529), was first reported in November 2021 in South Africa. Due to its high transmissibility and immune evasion, it rapidly replaced the previous Delta variant as the dominant variant. Omicron spreads quickly in multiple countries and has resulted in over 50% of confirmed COVID‐19 cases according to the World Health Organization (WHO).^1^ During the prevalence of Omicron, it gradually evolves into several sublineages, some of which have been dominant worldwide. Recently, two new Omicron subvariants, namely, BQ.1 and XBB, progressively replaced other Omicron subvariants in numerous countries and raised global concerns. It is worth noting that BQ.1, XBB, and their sublineages have demonstrated increased transmissibility and immune escape potential. Uraki et al.^2^ have found that BQ.1.1 and XBB effectively evade current humoral immunity elicited by COVID‐19 mRNA vaccines or natural infection. Moreover, BQ.1, XBB, and their sublineages show more resistance to therapeutic monoclonal antibodies (mAbs) than previous variants, even compared with earlier Omicron subvariants.^3^ Accordingly, these findings raise concerns about the risk of a new wave of COVID‐19.

In this study, we summarized the epidemiological and clinical characteristics of the BQ.1 and XBB lineages and also described their ability to escape from the neutralization of COVID‐19 vaccinated, convalescent sera, or mAbs. In addition, possible strategies for preventing the spread of these lineages and future SARS‐CoV‐2 variants are also highlighted.

## 2 EPIDEMIOLOGICAL FEATURES OF BQ.1 AND XBB LINEAGES

### 2.1 Origin and spread of BQ.1 and XBB lineages

**FIGURE 1:**
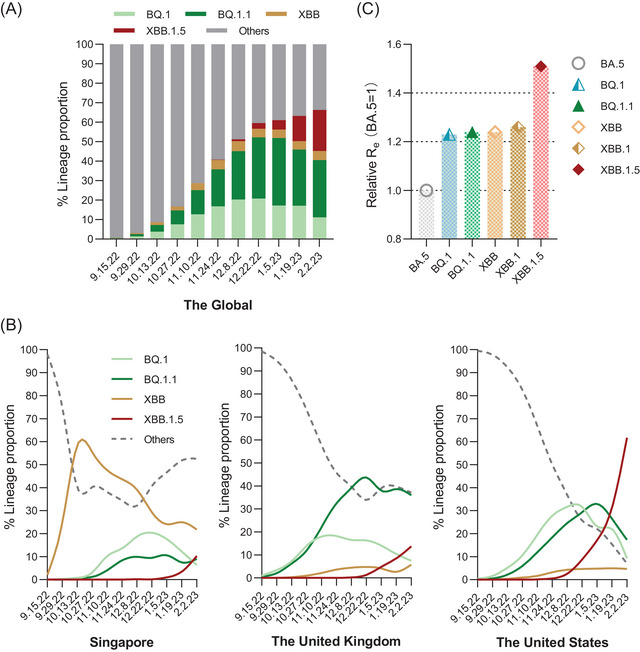
The estimated proportions of BQ.1 and XBB lineages. (A) and (B) show the proportions of the Omicron subvariants and other variants that moved every two weeks from 15 September, 2022 to 2 February, 2023 globally and in other three countries (Singapore, USA and UK). Data were obtained from GISAID and last accessed on 2 February 2023. (C) shows the global Re of BA.5, BQ.1, BQ.1.1, XBB, XBB.1 and XBB.1.5.

Omicron subvariant BQ.1 is an offshoot of BA.5. The first documented confirmed infection by BQ.1 could date back to February 2022 according to the WHO.^4^ BQ.1.1, a descendant of BQ.1, emerged several months later. Owing to their enhanced transmissibility, BQ.1 and BQ.1.1 expanded dramatically and replaced the previously dominating BA.5 in numerous countries. In the middle of September, the proportion of infections related to BQ.1 and BQ.1.1 was only approximately 2% of global diagnosed cases, but this proportion increased to 52.22% in early December 2022 (Figure 1A). BQ.1 and BQ.1.1 comprised 60.92% and 60% of infections in the United States and the United Kingdom in the middle of December 2022, respectively (Figure 1B). Afterward, they contributed to over 50% of infections around the world in January 2023 (Figure 1A).

XBB, the latest Omicron circulating subvariant, is a recombinant of BA.2.10.1 and BA.2.75 variants and further drives descendent lineages XBB.1 and XBB.1.5.^4^ In the first week of October 2022, XBB had a global prevalence of 1.3% and spread to 35 countries.^5^ Then, XBB dominated in several countries. Infections caused by XBB have been rising sharply and made up about 60% of cases in Singapore at the peak of the XBB wave. In contrast, XBB.1.5 spread quickly in the USA. As of 18 February 2023, the Centers for Disease Control and Prevention (CDC) estimated that XBB.1.5 accounted for 80.2% of new infections in the USA.^6^ According to WHO reports, 8931 sequences of XBB.1.5 subvariants were reported from 54 countries from 22 October 2022 to 23 January 2023, but over 75% of sequences were submitted by the USA.^7^ As of 21 February 2023, the XBB.1 and XBB.1.5 have spread to more than 100 countries.^8^

### 2.2 Clinical features of the BQ.1 and XBB lineages

Bayesian analysis has demonstrated that the effective reproduction numbers (*R*_e_) of BA.4/5 are higher than BA.2.^9^ Nevertheless, when the *R*_e_ value of BA.5 is set at 1, the relative *R*_e_ values of XBB and XBB.1 are 1.24 and 1.26, and those of BQ.1 and BQ.1.1 are 1.23 and 1.24, respectively (Figure 1C), suggesting that both BQ.1 and XBB are more effectively transmitted than BA.2 and BA.5.^10^ It is worth noting that the *R*_e_ of XBB.1.5 is more than 1.2‐fold greater than that of XBB.1, indicating that the transmission ability of XBB.1.5 is stronger than that of other variants, and more likely to cause a new pandemic.^11^

Data released by the Singapore Health Ministry revealed that the surge in confirmed cases of XBB infection during October 2022 did not result in higher mechanical ventilation, ICU admissions, or mortality.^12^ From 20 September 2022 to 17 October 2022, the risks for these outcomes were 0.03%, 0.2%, and 0.02%, respectively.^13^ Hospitalization of COVID‐19 patients infected with the XBB subvariant was even lower than that of patients infected with BA.5.^14^ Analysis of data released by the CDC revealed that the BQ.1 wave did not cause an increase in confirmed cases in October 2022. In contrast, following the emergence of XBB at the end of November 2022, there was an upward trend in confirmed cases, exacerbating hospital admissions.^15^ Nonetheless, the proportion of deaths did not escalate with the increased number of inpatients. Moreover, data from the New York government revealed that the number of ICU admissions attributed to BQ.1 and XBB was similar to previous BA.5 infections.^16^ These findings highlight that although the BQ.1 and XBB lineages have a transmission advantage over BA.5 and pose significant pressure on the global healthcare system, the risk of serious clinical outcomes from the BQ.1 and XBB lineages remains relatively low.

### 2.3 Spike mutations and infectivity of BQ.1 and XBB lineages

**FIGURE 2:**
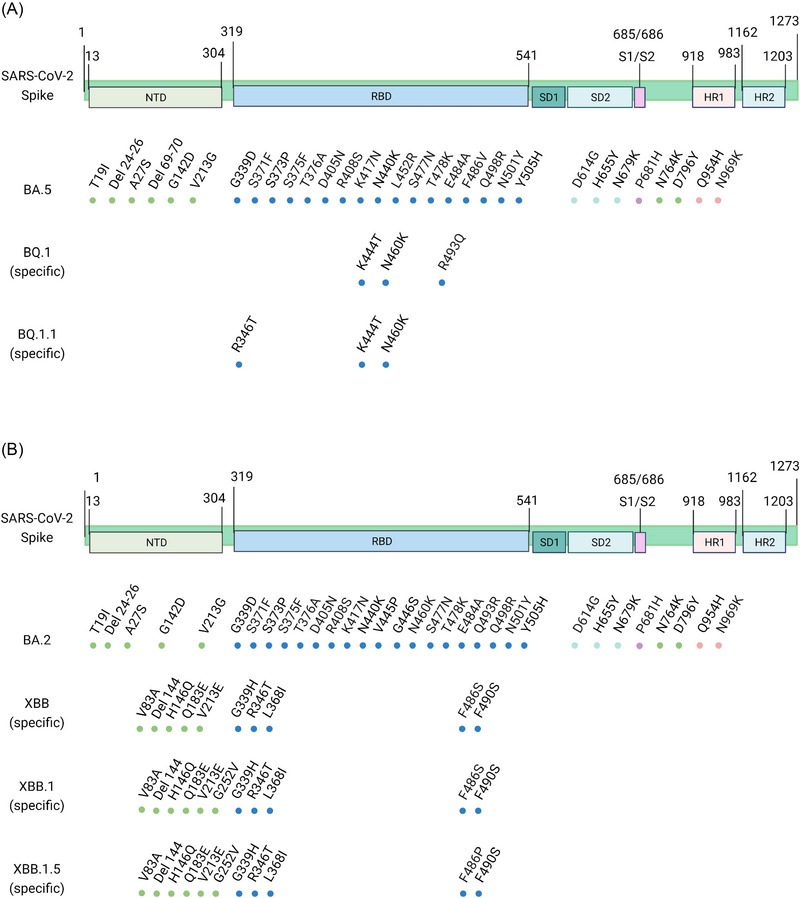
Schematic diagram shows the spike mutations of BQ.1 (A) and XBB lineages (B), including their ancestral strains BA.5 and BA.2. The mutations of BA.2, BA.5, BQ.1, and XBB are obtained from Refs. [3, 8, 59], and last accessed on 20 January 2023.

Current evidence suggests that BQ.1 and its subvariants are derived from BA.5. Therefore, as shown in Figure 2A, BQ.1 and BQ.1.1 share similar mutations in the spike protein of BA.5. BQ.1 has additional K444T, N460K, and Q493R mutations, whereas BQ.1.1 carries an additional R346T substitution. Mutations (such as L452R and F486V) account for twofolds increase in the infectivity of BA.5 compared to BA.2, resulting in the replacement of BA.2 by BA.5 in late June 2022.^17^ These mutations are inherited by BQ.1 and BQ.1.1 and further endow transmission advantages. The binding free energy (BFE) of BQ.1.1 RBD‐human angiotensin‐converting enzyme 2 (hACE2) complexes is greater than 4.0 kcal/mol, which is higher than that of BQ.1 and BA.5.^17^ This indicates its high potential to replace BA.5 as a new dominant variant, consistent with the recent fast spread of BQ.1.1. For the recombinant XBB subvariants, a large portion of the mutations in the spike protein is derived from BA.2. Intriguingly, it has evolved 10 new mutations, including V83A, H146Q, Q183E, V213E, G339H, R346T, L368I, F486S, and F490S substitutions and an amino acid deletion at site 144 (Figure 2B). XBB.1 and XBB.1.5 harbor one more G252V substitution than XBB, whereas XBB.1.5 carries an F486P substitution rather than the F486S substitution found in XBB and XBB.1 (Figure 2B). Compared to the ancestral strain, this rare substitution (F486P) is related to the RBD‐human angiotensin‐converting enzyme 2 (hACE2) binding affinity and transmissibility.^18^, ^19^ Uriu et al.^11^ found that the binding affinity of the XBB.1.5 RBD to hACE2 is 3.3‐fold higher than that of XBB.1. These results suggest that XBB.1.5 may outcompete XBB.1 and rapidly spread worldwide in the near future.

Some studies have assessed the infectivity and fusogenicity of XBB and BQ.1.1 subvariants through pseudovirus and cell fusion assays. Compared with BA.2, the infectivity of XBB and BQ.1.1 exhibited a 7.6‐ and 17‐fold increase, respectively, which resulted from the substitutions and deletions in XBB (V83A, R346T, L368I, and N460K) and BQ.1.1 (H69‐, V70‐, L452R, and F486V).^10^, ^20^ Similar to BA.2, the increased infectivity of XBB or BQ.1.1 is independent of TMPRSS2.^21^ In addition, the V83A, R346T, and N460K mutations in XBB and the R346T and N460K substitutions in BQ.1.1 account for their increased fusogenicity.^10^, ^20^ Moreover, in vivo experiments analyzed body weight, respiratory disorders, RNA loads (in the oral swab, lung hilum, and periphery), and histopathological lung features from hamsters that were infected with different strains. It is noticeable that the intrinsic pathogenicity of XBB was similar to or slightly lower than BA.2.75, whereas this trend was also observed in BQ.1.1 compared to BA.5.^20^, ^22^, ^23^

Overall, these findings may explain the higher transmissibility yet low risks of hospitalization and mortality observed during the BQ.1 and XBB outbreaks.

## 3 IMMUNE EVASION OF BQ.1 AND XBB LINEAGES

During the SARS‐CoV‐2 pandemic continuum, the virus has constantly evolved, leading to the emergence of variants with a progressive increase in transmissibility.^24^ In this evolutionary race, Omicron with substantial mutations was first documented in 2021.^25^ These mutations mainly located in the spike protein are critical for antibody binding and responsible for the sharp increase in infectivity and immune evasion.^26^, ^27^, ^28^ In this respect, the recently documented subvariants of Omicron (BQ.1 and XBB lineages) harbor an alarming number of spike protein mutations, leading to concerns about the efficacy of the current vaccines and mAbs. Hence, it is necessary to understand the mechanisms of immune evasion capacity, antibody recognition epitopes, and B‐cell epitopes of those new subvariants.^29^

### 3.1 Spike mutations and immune evasion

An increasing body of evidence suggests that spike mutations can alter RBD‐ACE2 interactions, increase infectivity, and restrict antibody‐virus recognition.^30^, ^31^ Homology modeling of the spike protein shows that the mutations (K444T, N460K, and R346T) do not enhance the interaction between ACE2 and spike protein compared to BA.5.^32^, ^33^, ^34^ However, K444T and R346T can reportedly alter the antibody recognition epitope and destabilize the affinity between RBD and related antibodies to exacerbate immune escape. These mutation‐mediated immune escape mechanisms are further explained by modeling the SARS‐CoV‐2 structure and constructing pseudoviruses. Although K444T and R346T were isolated from the receptor‐binding motif, they are located precisely in the epitopes of class III neutralizing antibodies, thus impairing the potency of class III mAbs.^35^ In addition, structural information suggests that R346T and K444T mediate antibody recognition by affecting the stability of hydrogen bonds and salt bridges.^35^ The loss of neutralizing activity of NTD‐SD2 and class I mAbs has been associated with the N460K mutation.^3^ Furthermore, subtle but non‐negligible modifications caused by these mutations in BQ.1 and BQ.1.1 potential B‐cell epitopes were observed, which might also contribute to the intensified immune evasion.^32^

Current evidence suggests that the V445P substitution in XBB sublineages confers the ability to evade antibodies directed to the spike regions by damaging hydrogen bonds and inducing steric hindrance.^3^ The F486S mutation is a critical residue embedded in the RBD that can bind to ACE2 and neutralize antibodies. It has been shown that F486S interacts with residues in the antibody to avoid the binding of classes I and II mAbs.^35^ Moreover, serum neutralization assays have corroborated that the F486S mutation significantly enhances resistance to neutralizing antibodies compared to R346T.^35^ Meanwhile, the additional mutation F490S in XBB/XBB.1/XBB.1.5 can abolish the cation‐*π* interaction and result in the loss of antibody neutralization.^3^ In addition to the RBD, the Y144del substitution in the NTD of the XBB lineage has been associated with robust immune evasion from NTD‐targeting neutralization antibodies.^36^, ^37^, ^38^, ^39^

### 3.2 Neutralization by therapeutic monoclonal antibodies

Currently available monoclonal antibodies for SARS‐CoV‐2 can be grouped into two main categories based on whether they can bind to the RBD. Antibodies that bind to the RBD can be further classified into four classes: classes I–III block ACE2 and bind to the RBD in different states (up, down, both up and down), whereas class IV is incapable of blocking ACE2 and binds only to “up” RBDs.^40^ It is widely thought that subvariants enhance the evasion ability of the virus from regulatory‐approved monoclonal antibodies.

The neutralization IC_50_ value is often applied to assess the resistance of SARS‐CoV‐2 variants against monoclonal antibodies. In a study, 24 antibodies were included and tested for neutralizing efficacy, consisting of 4 class I monoclonal antibodies, 4 class II antibodies, 11 class III antibodies, 1 class IV antibody, 4 antibodies that do not bind the RBD and Evusheld (Table 1).^3^ The fold change in IC_50_ revealed that 19 antibodies had poor activity against BQ.1 and BQ.1.1 compared to BA.4/5. The most significant decrease was observed with LY‐CoV1404, with a 7668‐fold decrease in BQ.1/BQ.1.1. Similarly, XBB and XBB.1 exhibit a highly similar and alarming ability to escape from all antibodies except for S3H3, which means that these monoclonal antibodies are defenseless against the attack of XBB and XBB.1.

**TABLE 1:** Resistance to D614G, BQ.1/BQ.1.1 and XBB/XBB.1/XBB.1.5 by different monoclonal antibodies.

Pseudovirus IC_50_ (ng/mL)	Sotrovimab	Bebtelovimab	Imdevimab	Casirivimab	Cilgavimab	Tixagevimab	SA55	SA58	SA55 + SA58	Imdevimab + Casirivimab	Cilgavimab + Tixagevimab	Reference
D614G	23‐74	0.7‐2	2.1‐5.7	1.7‐5.6	2.5‐7	1.3‐2	11	0.9‐2	2.1	1.6‐5	1.3‐3	3, 41, 46
BA.2	833‐918^a^	0.9‐4.5	112‐590	>10,000	6.1‐9	1,924‐4,312	7.2	5.1‐14	7.8	821‐1,985	8.2‐24.7	3, 36,41 46
	>10,000^b^											
BA.4/5	514‐1,088	0.8‐2	208‐520	>10,000	11‐23	>10,000	5	3.9‐9	4.5	700‐709	35‐40	3, 36, 41, 46
BQ.1	600‐1,709	>10,000^#^	>10,000	>10,000	>10,000	>10,000	6.6	34‐44	9.2	>10,000	>10,000	3, 36
		1,905^*^										
BQ.1.1	2,140‐7,640	>10,000	>10,000	>10,000	>10,000	>10,000	5.9	>10,000^#^	10	>10,000	>10,000	3, 36, 41
								900^*^				
XBB	343‐963	>10,000	>10,000	>10,000	>10,000	>10,000	5.3	>10,000^#^	9.8	>10,000	>10,000	3, 36
								8,805^*^				
XBB.1	405‐896	>10,000	–	–	>10,000	>10,000	6.2	>10,000	12	–	>10,000	3, 19
XBB.1.5	915	>10,000	–	–	–	–	7.1	>10,000	11	–	>10,000	19

^a^represents ref. 3, ref. 36 and ref. 46; ^b^represents ref. 41; ^#^represents ref. 3; ^*^represents ref. 36.

In addition, emerging evidence suggests that the monoclonal antibodies Imdevimab, Casirivimab, Amubarvimab, Romlusevimab, Regdanvimab, Bamlanivimab, Etesevimab, Adintrevimab, and DXP‐604 exhibit decreased therapeutic effects against BQ.1/BQ.1.1 and XBB (Table 1).^36^, ^41^, ^42^, ^43^ Moreover, failure to neutralize BQ.1.1 and XBB has been observed with cocktail therapies, such as Imdevimab‐Casirivimab, Amubarvimab‐Romlusevimab, and Bamlanivimab‐Etesevimab.^36^, ^42^, ^43^ Evusheld and LY‐CoV1404, two promising and highly recommended antibodies, have been reported to exhibit tremendous neutralization abilities against most SARS‐CoV‐2 variants, including Omicron BA.1, BA.2, BA.4, and BA.5, but are significantly affected by the XBB.1.5 pseudovirus.^19^, ^44^, ^45^

It has been established that the potency of some mAbs against BQ.1 and XBB is maintained. For example, SA58 mAb exhibits high potency against BQ.1 and BQ.1.1, whereas SA55 displays high neutralization efficacy against both BQ.1 and XBB subvariants (Table 1).^3^, ^19^, ^46^ Even the SA55‐SA58 cocktail has been reported to induce appreciable neutralization potency of BQ.1 and XBB lineages (Table 1).^19^, ^36^ The resistance to D614G, BQ.1, and XBB lineages by different monoclonal antibodies has been summarized in Table 1.

### 3.3 Ability to breakthrough COVID‐19 vaccine‐induced immunity

The perpetual emergence of new subvariants has raised the alarm about the risk of escape from the vaccine‐elicited antibodies. Sera from healthcare workers who had received three doses of mRNA vaccine exhibited extreme neutralizing resistances against the BQ.1 and BQ.1.1 subvariants. Analysis of the neutralization titers revealed a 10.6‐, 11‐, 18.7‐, and 22.9‐fold reduction in the ability of serum antibodies against BA.4/5, BF.7, BQ.1, and BQ.1.1, respectively, compared to D614G (Table 2).^35^ Likewise, the 3‐dose CoronaVac vaccine was associated with substantially reduced neutralization ability against BQ.1, BQ.1.1, XBB, and XBB.1 (Table 2).^36^

**TABLE 2:** Geometric mean 50% neutralizing antibody titer (NT_50_ GMT) of different vaccines against D614G and Omicron subvariants.

NT_50_ GMT	D614G	BA.2	BA.4/5	BQ.1	BQ.1.1	XBB	XBB.1	XBB.1.5	Reference
3 doses BNT162b2 or mRNA‐1273	257	31.2	20.3	–	12.2	11.9	–	–	2
	2616	759	300	140	114	–	–	–	35
	1445	475	197	–	25	43	42	59	54
4 doses BNT162b2 or mRNA‐1273	727	88	62.2	–	16.8	14.1	–	–	2
	1533	–	95	–	22	–	15	–	49
3 doses ChAdOx1	652	130	72	27	24	20	20	–	36

Moreover, analysis of the protective effect induced by different doses of the BNT162b2 or mRNA‐1273 vaccine against BQ.1/BQ.1.1 and XBB/XBB.1 showed that the immune effect induced by three or four doses was modest and almost undetectable (Table 2).^2^, ^3^ During the Omicron pandemic, Moderna developed a bivalent vaccine aimed at Omicron; thus, the effectiveness of this bivalent BA.5 vaccine against BQ.1 and XBB is also a matter of concern.^47^, ^48^ A study collected sera from volunteers who received four doses of BNT162b2/mRNA‐1273 vaccine or a BA.5 bivalent booster to determine the neutralizing activities against BQ.1.1 and XBB.1 (Table 2).^49^ It was found that the four doses of mRNA vaccine could not provide effective prophylaxis against new Omicron subvariants. The XBB.1 subline was associated with the lowest geometric mean 50% neutralizing antibody titer (NT_50_ GMT), below the detection limit of the assay (<20), followed by BQ.1.1. However, the BA.5 bivalent booster induces a definite but not significant increase in the neutralizing capacity against all Omicron descendants with NT_50_ GMTs of 298, 305, 183, 98, 73, and 35 against BA.4/5, BF.7, BA.4.6, BA.2.75.2, BQ.1.1, and XBB.1, respectively, whereas this value in WT is 3620.^49^ Similarly, the bivalent booster seems more effective against XBB than other vaccines, but the margin of improvement in immunity is not significant.^50^ A study assessed the immune response of participants who received bivalent boosters at three different time points and found that both XBB and XBB.1.5 were able to evade neutralizing antibody (NAb) responses, but not T cell responses. NAb titers to XBB.1 and XBB.1.5 were similar, indicating that the F486P mutation in XBB.1.5 may increase its transmissibility but does not enhance its ability to evade the immune system.^51^

As mentioned earlier, the emerging subvariants of Omicron display an increased immune evasion tendency, which enables the virus to escape antibody immunity induced by current vaccines, including boosters. Among them, XBB lineages show the highest degree of immune evasion. Data on vaccine effectiveness are summarized in Table 2.

### 3.4 Resistance to convalescent sera

Although convalescent individuals produce serum antibodies against SARS‐CoV‐2, BQ.1, and XBB can still exhibit immune evasion. A study explored whether infection with different SARS‐CoV‐2 variants could protect against reinfection. Serum samples from individuals with Delta, BA.1, BA.2.2, BA.5.12, and BA.2.76 infections revealed that BQ.1 and BQ.1.1 yielded different degrees of immune escape from neutralizing antibodies.^52^ For example, in the BA.1 breakthrough infection group, the neutralization sensitivity of BQ.1 was markedly reduced, and only 47.4% of serum samples could neutralize BQ.1.1 compared with BA.2 (94.7%) and BA.2.75 (100%).^52^ Consistently, another study analyzed several Omicron variants using sera from infected cases during the BA.1 wave and BA4/5 wave. During both waves, BQ.1 and BQ.1.1 demonstrated robust immune escape abilities, significantly higher than other variants.^35^ Convalescent individuals who received three doses mRNA vaccine and were later infected with BA.2 exhibited a significantly reduced ability to neutralize the BQ.1.1 and XBB subvariants, with a 35.2‐ and 61.7‐fold decrease, respectively, compared to those against the ancestral strain.^2^ Moreover, XBB.1 was associated with a higher evasion capacity than wild‐type in patients treated with different doses of vaccines and infection history.^53^ The neutralization titers against XBB.1.5 were evaluated using the sera from convalescent who had received three doses of CoronaVac vaccine before being infected with the D614G, BA.5, or BF.7 variants.^19^ The results showed that CoronaVac provided excellent protection against the wild‐type virus and other variants. However, a significant reduction in neutralization titers was observed for XBB.1 and XBB.1.5 in all breakthrough infections. For the subgroups infected with BA.5 or BF.7 after CoronaVac, the NT50 decreased by 31–50 folds in XBB.1 and 27–40 folds in XBB.1.5. This trend was also seen in other breakthrough groups, indicating that XBB.1.5 had a slightly lower ability to evade the human immune response compared to XBB.1.^11^, ^54^

The previous studies overlap in their assertion that BQ.1 and XBB harbor greater immune escape capacity than the other Omicron variants in cases of breakthrough infections.

## 4 STRATEGIES FOR PREVENTION OF BQ.1 AND XBB SUBLINEAGES

It is widely thought that BQ.1 and XBB sublineages confer an immune evasion advantage over other circulating Omicron sublineages, suggesting a higher reinfection risk. Specifically, although the immune evasion abilities of XBB.1 and XBB.1.5 are comparable, they remain stronger than those of BQ.1/BQ.1.1. However, the increased transmissibility of XBB.1.5 is due to the enhanced receptor‐binding affinity mediated by F486P substitution.^19^ These results suggest that XBB.1.5 is the most advantageous XBB lineage as of January 2023, attributed to its strengthened receptor‐binding affinity without any significant loss in immune resistance. Consequently, this high growth advantage of XBB.1.5 may drive a new infection wave in the future.

Under such circumstances, wearing a mask and maintaining social distance remain effective measures to prevent infection by Omicron sublineages. As circulating Omicron subvariants, such as BQ.1.1 and XBB.1.5, are resistant to most clinically used mAbs, treatment with mAbs alone is ineffective, and broadly neutralizing monoclonal antibodies are urgently needed for COVID‐19 treatment.^43^ Indeed, other treatment options (e.g., paxlovid) should be considered against BQ.1.1 and XBB.1.5. Excellent inhibitory activity has been observed for three antiviral inhibitors called remdesivir, molnupiravir, and nirmatrelvir, authorized for emergency use in the USA, against both BQ.1.1 and XBB in vitro.^42^ Among the three drugs, the IC_50_ values of molnupiravir and nirmatrelvir were slightly higher for BQ.1.1 but 0.6‐fold lower for remdesivir than for the ancestral strain. XBB has been reported to exhibit mild tolerance to nirmatrelvir, whereas the other two drugs yielded a good therapeutic effect. A recent study utilized a technology called STage‐Enhanced Maturation (STEM) to select new therapeutic antibodies. They engineered seven broadly neutralizing antibodies that are highly effective against all tested variants of SARS‐CoV‐2, including XBB.1.5 and BQ.1.1.^55^ Meanwhile, Zhao et al.^56^ designed a new pan‐vaccine antigen (*S*_pan_) based on the diverse evolution of SARS‐CoV‐2 spike sequences. It was found that S_pan_ vaccination of mice elicited broad immunity to several variants, including Omicron BA.1. Those methods may combat SARS‐CoV‐2 antigenic drift and confer a rapid development of broadly neutralizing therapeutic mAbs.

Original spike‐based vaccines exhibit a mitigated protective effect against new Omicron subvariants. The present literature suggests that the probability of severe COVID‐19 and dyspnea in vaccinated people is lower than in unvaccinated people, which indicates that no major impact on vaccination protection against severe disease and death is foreseen even against BQ.1.1 and XBB.1.5.^57^, ^58^ Therefore, getting vaccinated should still be encouraged before the licensing of specific vaccines against BQ.1.1 and XBB.1.5 or broad‐spectrum vaccines.

## 5 CONCLUSION AND PERSPECTIVE

**FIGURE 3:**
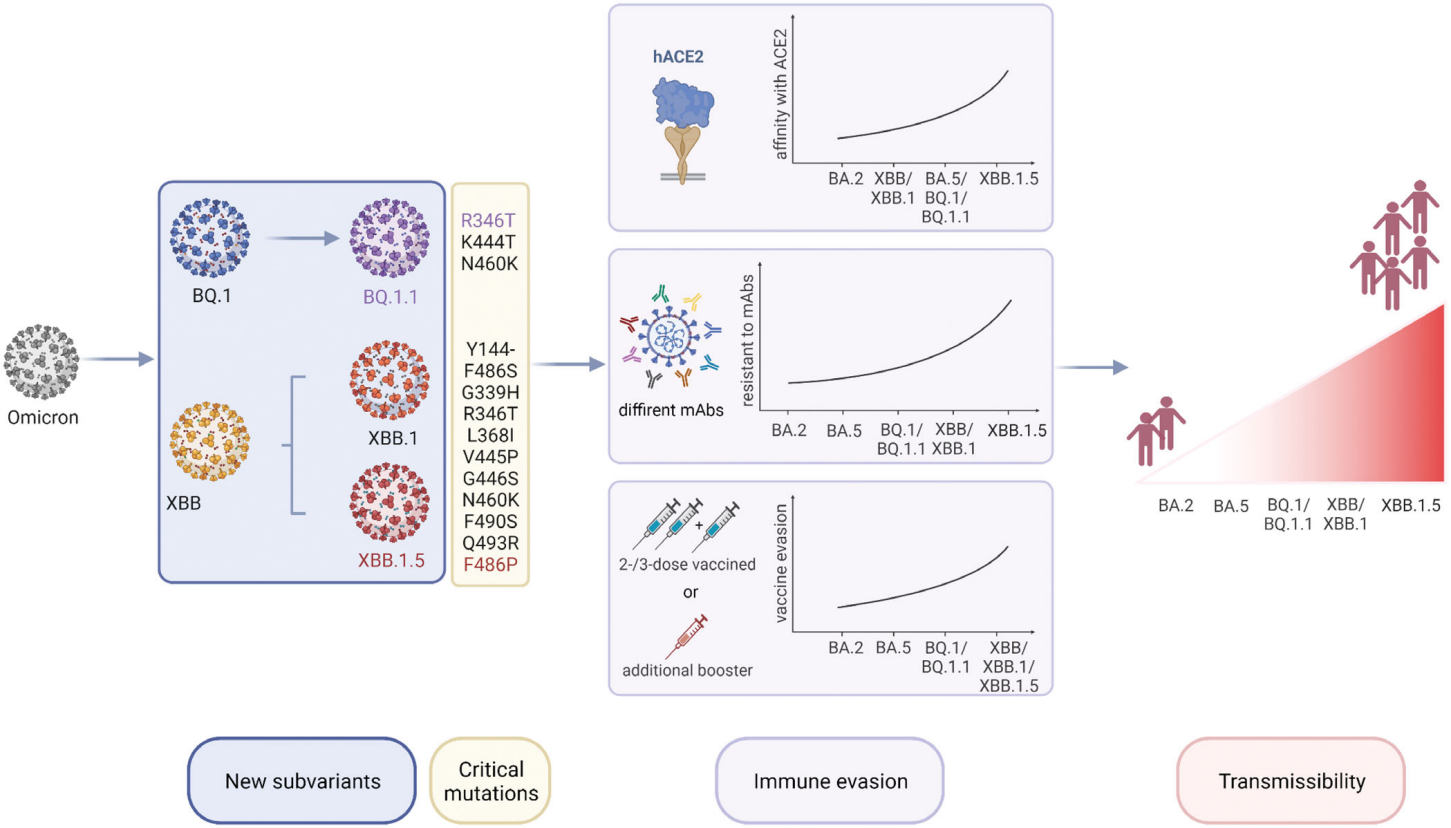
The superior transmissibility of BQ.1 and XBB lineages. New subvariants of the Omicron variant, BQ.1/BQ.1.1 and XBB/XBB.1/XBB.1.5, with substantial mutations, have contributed to the advantage in immune evasion and sharp increase in infectivity.

During the ongoing COVID‐19 pandemic, SARS‐CoV‐2 is constantly evolving and mutating. The more recently emerged subvariants of the Omicron variant, BQ.1 and XBB, have quickly become a global public health issue with the ability to limit the effectiveness of the available vaccines and therapeutic monoclonal antibodies, causing a growth advantage (Figure 3). In this case, it is still necessary to promote global vaccination with existing vaccines and emphasize the importance of boosters, particularly among the elderly and children.

Moreover, representative testing and genomic surveillance of each SARS‐CoV‐2 variant are pivotal to identifying novel variants and predicting their immune escape capacity, which is essential to optimize the formulation of mRNA vaccines, evaluate broadly active clinical monoclonal antibodies, and guide public health authorities in taking appropriate prevention and control measures. In addition, maintaining a high level of immunity, avoiding reinfections, and reducing the risk of long COVID are equally crucial for all age groups. Furthermore, governments should provide timely warnings and optimize pandemic controls based on domestic conditions to prevent coinfection with other infectious diseases, such as seasonal influenza. More studies are warranted to obtain optimal approaches for epidemic prevention and therapeutic strategies against future COVID‐19 outbreaks.

## AUTHOR CONTRIBUTIONS

Xiawei Wei conceived the study and revised the manuscript. Danyi Ao, Xuemei He, and Weiqi Hong wrote the paper. All authors have read and approved the final manuscript.

## CONFLICT OF INTEREST STATEMENT

The authors declare no conflict of interests.

## ETHICS STATEMENT

Not applicable.

## Data Availability

The data included in this study are available upon request from the corresponding author.
